# *QKI* Promotes Sheep Preadipocyte Differentiation by Reducing *Cavin3* mRNA Stability

**DOI:** 10.3390/ani16152441

**Published:** 2026-08-06

**Authors:** Zicheng Li, Changsong Xu, Bokang Shan, Yuan Wang, Wangyang Qin, Lei Xia, Liying Qiao, Wenzhong Liu, Yangyang Pan

**Affiliations:** 1 College of Animal Science, Shanxi Agricultural University, Jinzhong 030801, China; 202430624@stu.sxau.edu.cn (Z.L.); 20232408@stu.sxau.edu.cn (C.X.); 202420341@stu.sxau.edu.cn (B.S.); wangyuan39@sxau.edu.cn (Y.W.); qinwangyang1@sxau.edu.cn (W.Q.); liyingqiao@sxau.edu.cn (L.Q.); wenzhongliu@sxau.edu.cn (W.L.); 2 Shanxi Provincial Key Laboratory of Livestock and Poultry Genetic Resources Exploration and Biotechnology Breeding, Shanxi Agricultural University, Jinzhong 030801, China; 3 Faculty of Software Technologies, Shanxi Agricultural University, Jinzhong 030801, China; xialei@sxau.edu.cn

**Keywords:** quaking, caveolae-associated protein 3, sheep preadipocytes, adipogenic differentiation, RNA-binding protein, mRNA stability, glucose consumption, full-length transcriptome sequencing

## Abstract

Adipogenic differentiation is essential for adipose tissue development, fat deposition, and metabolic regulation in livestock, but the role of RNA-binding proteins in sheep preadipocyte differentiation remains unclear. This study investigated whether Quaking (*QKI*) regulates sheep preadipocyte adipogenesis and explored its downstream regulatory mechanism. *QKI* expression was examined in sheep adipose tissues, and *QKI* knockdown was performed in sheep preadipocytes. Adipogenic marker expression, lipid accumulation, glucose consumption, Oxford Nanopore Technologies (ONT) full-length transcriptome sequencing, RNA immunoprecipitation coupled with quantitative real-time PCR (RIP-qPCR), actinomycin D assay, caveolae-associated protein 3 (*Cavin3*) knockdown, and simultaneous *QKI* and *Cavin3* knockdown were used to evaluate the function and mechanism of *QKI*. *QKI* was expressed in different sheep adipose tissues, with relatively higher expression in tail fat. *QKI* knockdown reduced adipogenic marker expression, Oil Red O staining, and glucose consumption after adipogenic induction. ONT full-length transcriptome sequencing identified *Cavin3* as a markedly upregulated candidate downstream target after *QKI* knockdown. RIP-qPCR showed enrichment of *Cavin3* mRNA in QKI immunoprecipitates, and actinomycin D assays indicated that *QKI* knockdown delayed *Cavin3* mRNA degradation. *Cavin3* knockdown enhanced adipogenic marker expression, Oil Red O staining, and glucose consumption. Simultaneous *QKI* and *Cavin3* knockdown increased adiponectin, fatty-acid-binding protein 4, and peroxisome proliferator-activated receptor gamma mRNA abundance and Oil Red O staining relative to the matched control, whereas glucose consumption was not significantly different. These findings suggest that *QKI* promotes sheep preadipocyte differentiation, at least in part, by reducing *Cavin3* mRNA stability, providing insight into RNA-binding protein-mediated regulation of livestock adipogenesis.

## Simple Summary

Fat formation in sheep is important for animal growth, meat quality, and energy metabolism, but the biological processes that control how sheep fat cells develop are not fully understood. This study investigated how Quaking (QKI), a gene-regulating protein, affects the development of sheep precursor fat cells into mature fat cells. We found that reducing QKI levels weakened fat cell formation, decreased Oil Red O staining, and reduced glucose use by the cells. Further analysis showed that *QKI* was associated with caveolae-associated protein 3 (*Cavin3*) messenger RNA and affected its stability. When *Cavin3* was reduced, fat cell formation and glucose use increased, showing an effect opposite to that caused by *QKI* reduction. An independent combined *QKI* and *Cavin3* knockdown experiment further supported the involvement of *Cavin3* in *QKI*-regulated fat cell development. These results suggest that *QKI* helps sheep fat cells develop partly through the post-transcriptional regulation of *Cavin3*. This study improves our understanding of how fat development is controlled in sheep and may provide useful information for future research on livestock growth, meat quality, and metabolic regulation.

## 1. Introduction

Adipose tissue is not only a passive site for energy storage but also a dynamic metabolic and endocrine organ that coordinates lipid handling, glucose utilization, insulin responsiveness, and systemic energy homeostasis [1,2]. In livestock species, the developmental and metabolic state of adipose tissue is closely related to growth efficiency, carcass composition, fat distribution, and meat quality [3,4,5]. Sheep exhibit distinct patterns of fat deposition among different adipose depots, including subcutaneous, visceral, intramuscular, and tail fat [6,7]. These depot-specific characteristics provide an informative biological context for investigating the molecular mechanisms that govern adipocyte differentiation and fat accumulation.

Adipogenic differentiation is a coordinated process in which preadipocytes acquire mature-adipocyte morphology and metabolic function [8]. This transition is governed by core regulators, including peroxisome proliferator-activated receptor gamma (PPARγ), CCAAT/enhancer-binding protein alpha (C/EBPα), fatty-acid-binding protein 4 (FABP4), and adiponectin (ADIPOQ), together with remodeling of lipid synthesis, glucose metabolism, the cytoskeleton, and membrane trafficking [9,10]. Beyond transcriptional control, post-transcriptional regulation determines the timing, magnitude, and persistence of adipogenic gene expression [11]. RNA-binding proteins can regulate mRNA splicing, localization, translation, and degradation, thereby influencing differentiation and metabolic remodeling [12].

Quaking (*QKI*) is a conserved member of the signal transduction and activation of RNA (STAR) family of RNA-binding proteins [13]. Through association with target RNAs, *QKI* can influence transcript processing and stability in a context-dependent manner [14,15,16]. *QKI* has been implicated in cell differentiation [17], tissue development, and adipose-tissue metabolism [18], but its role and candidate RNA targets during sheep preadipocyte differentiation remain unclear. Caveolae-associated proteins link membrane architecture, signal transduction, and metabolic function [19]. Caveolae are abundant in adipocytes and participate in lipid trafficking, insulin signaling, membrane organization, and cellular stress responses [20]. Caveolae-associated protein 3 (*Cavin3*) contributes to caveolar dynamics and has been linked to adipocyte differentiation in mouse, human, and porcine models [21,22]. Whether *Cavin3* has the same role in sheep preadipocytes and whether it is post-transcriptionally regulated by *QKI* are unknown.

We hypothesized that *QKI* supports sheep preadipocyte differentiation partly by regulating *Cavin3* mRNA stability. The objectives were to (i) characterize *QKI* abundance in sheep adipose depots and determine the effect of *QKI* knockdown on adipogenic differentiation; (ii) identify *QKI*-responsive candidate transcripts by ONT full-length transcriptome sequencing; (iii) test the association of *QKI* with *Cavin3* mRNA and evaluate *Cavin3* mRNA decay after *QKI* knockdown; and (iv) determine whether *Cavin3* knockdown, alone and together with *QKI* knockdown in an independent experiment, produced phenotypes consistent with *Cavin3* contributing to *QKI*-dependent adipogenesis.

## 2. Materials and Methods

### 2.1. Isolation and Culture of Sheep Stromal Vascular Fractions

Subcutaneous adipose tissue was collected from 3-day-old male Dorper × Hu *F*_1_ lambs (*n* = 3) for stromal vascular fraction (SVF) isolation. All animal procedures were approved by the Animal Ethics Committee of Shanxi Agricultural University (SXAUEAW2022S.NO.004010001). For adipose-depot expression analysis, subcutaneous, tail, and visceral adipose tissues were collected from the same batch of lambs and immediately processed or snap-frozen in liquid nitrogen. The adipose tissue was first immersed in ice-cold 75% ethanol for 1 min and then transferred to ice-cold phosphate-buffered saline (PBS; KGL2206, KeyGEN, Nanjing, China) supplemented with 1% penicillin–streptomycin (15140122, Gibco, Grand Island, NY, USA).

Subcutaneous adipose tissues from the three lambs were pooled before SVF isolation. The collected adipose tissue was cut into approximately 1 cm^3^ pieces and placed into centrifuge tubes containing 2 mg/mL collagenase type I (17100017, Gibco, Grand Island, NY, USA). The tissue was further minced and digested at 37 °C for 40 min, with vigorous shaking every 5 min to ensure uniform digestion. Digestion was terminated by adding an equal volume of complete medium consisting of high-glucose Dulbecco’s Modified Eagle’s Medium (DMEM; KGL1211, KeyGEN, Nanjing, China), 10% fetal bovine serum (FBS; KGL3006, KeyGEN, Nanjing, China), and 1% penicillin–streptomycin.

The digested cell suspension was centrifuged at 500× *g* for 5 min. The pellet was resuspended in complete medium and filtered sequentially through 100-μm (258367, NEST, Wuxi, China) and 40-μm (258369, NEST, Wuxi, China) cell strainers. Cells were seeded in 10-cm culture dishes (704202, NEST, Wuxi, China) and maintained at 37 °C with 5% CO_2_ in a humidified incubator (Thermo Fisher Scientific, Waltham, MA, USA). The culture medium was replaced every 24 h.

### 2.2. Adipogenic Induction of Sheep Preadipocytes

When sheep preadipocytes reached confluence, they were cultured for an additional 24 h and induced with high-glucose DMEM containing 10% FBS, 1% penicillin–streptomycin, 1.8 μg/mL bovine insulin (I8040, Solarbio, Beijing, China), 0.51 μM dexamethasone (D8040, Solarbio, Beijing, China), 38 μM 3-isobutyl-1-methylxanthine (IBMX; I8450, Solarbio, Beijing, China), and 2 μM rosiglitazone (R8470, Solarbio, Beijing, China). Insulin, dexamethasone, and rosiglitazone have previously been used to support differentiation of sheep preadipocytes [23]. The induction medium was replaced every 2 days. After 4 days, it was replaced with maintenance medium containing DMEM, 10% FBS, 1% penicillin–streptomycin, 1.8 μg/mL bovine insulin, and 2 μM rosiglitazone; maintenance medium was replaced every 2 days.

### 2.3. Lentiviral-Mediated QKI Knockdown

The lentiviral interference vector targeting QKI and the corresponding negative control lentivirus were constructed and provided by Haixing Biosciences (Suzhou, China). Sequences are shown in Supplementary Table S1. Sheep preadipocytes were seeded into 6-well plates and infected when cell confluence reached 60–70%. Lentivirus was added at a multiplicity of infection (MOI) of 5 in the presence of polybrene (C0351, Beyotime, Shanghai, China) at a final concentration of 5 μg/mL. After 13 h of infection, the culture medium was replaced with fresh complete medium, and the cells were cultured for an additional 48–72 h to evaluate infection efficiency. QKI knockdown efficiency was assessed by quantitative real-time PCR (qRT-PCR).

### 2.4. siRNA-Mediated Cavin3 Knockdown

Small interfering RNAs (siRNAs) targeting *Cavin3* were synthesized by Sangon Biotech (Shanghai, China). Three specific siRNA sequences targeting *Cavin3* were designed, and a negative control siRNA was used in parallel. The siRNA sequences are listed in Supplementary Table S2.

Twenty-four hours before transfection, the culture medium was replaced with antibiotic-free complete medium. When cell confluence reached approximately 80%, siRNA transfection was performed according to the manufacturer’s instructions for KeygenMAX 3000 transfection reagent (KGA9705, KeyGEN, Nanjing, China). Briefly, siRNA and KeygenMAX 3000 reagent were separately diluted in Opti-MEM reduced-serum medium (MF988-01, Mei5bio, Beijing, China) and incubated at room temperature for 5 min. The diluted siRNA and transfection reagent were then gently mixed and incubated for another 15 min to allow the formation of transfection complexes. The complexes were subsequently added to the cell culture system.

The final concentration of siRNA was 40 nM. After 6 h of transfection, the medium was replaced with fresh antibiotic-free complete medium. Cells were collected 56 h after transfection, and *Cavin3* expression was measured by qRT-PCR to verify knockdown efficiency.

### 2.5. Combined QKI and Cavin3 Knockdown

An independent combined-knockdown experiment was performed using the *QKI*-targeting lentivirus and *Cavin3*-targeting siRNA described in Section 2.3 and Section 2.4, together with their corresponding negative controls. Knockdown of both targets was verified by qRT-PCR, after which cells were induced to differentiate. Adipogenic-marker mRNAs, qualitative Oil Red O staining, and glucose consumption were evaluated. This experiment was performed in a separate batch from the original QKI single-knockdown experiment; therefore, the double-knockdown group was compared only with its matched negative control and was interpreted as independent pathway-supporting evidence rather than as a quantitative measurement of complete rescue.

### 2.6. Oil Red O Staining

Lipid droplets were stained using an Oil Red O kit (G1262, Solarbio, Beijing, China) according to the manufacturer’s instructions. Cells were washed three times with PBS, fixed for 30 min, washed twice with distilled water, treated with 60% isopropanol for 20 s, stained with freshly prepared Oil Red O working solution at 37 °C for 20 min, rinsed with 60% isopropanol for 15 s, and washed five times with distilled water. Representative fields were photographed using an inverted microscope (DMi8, Leica Microsystems, Wetzlar, Germany).

### 2.7. RNA Extraction and Quantitative Real-Time PCR

Total RNA was extracted using the TRIzol method. After removal of the culture medium, cells were washed three times with pre-cooled PBS and lysed with 750 μL TRIzol reagent (9109, TAKARA, Shiga, Japan). The lysates were thoroughly pipetted to ensure complete lysis. Chloroform was added, followed by vortexing for 10 s and incubation for 10 min. The samples were centrifuged at 12,000× *g* at 4 °C, and the aqueous phase was collected. An equal volume of isopropanol was added to precipitate RNA. After centrifugation at 12,000× *g* at 4 °C, the RNA pellet was collected, washed three times with 75% ethanol, and briefly air-dried at room temperature. After complete evaporation of ethanol, RNA was dissolved in RNase-free water (R1600, Solarbio, Beijing, China).

RNA concentration and purity were measured using a NanoDrop 2000 spectrophotometer (Thermo Fisher Scientific, Waltham, MA, USA). Samples with A260/A280 ratios of 1.8–2.1 were reverse-transcribed using 1 μg total RNA and ToloScript All-in-one RT EasyMix (22107, Tolobio, Shanghai, China). qRT-PCR was performed on a CFX Connect Real-Time PCR Detection System (Bio-Rad, Hercules, CA, USA) using 2× Q5 SYBR qPCR Master Mix (21808, Tolobio, Shanghai, China). 

Each 10 μL qRT-PCR reaction contained 5 μL 2× SYBR qPCR Master Mix, 0.4 μL each of forward and reverse primers (10 μM), 2 μL cDNA, and ddH_2_O. Cycling comprised 95 °C for 30 s followed by 39 cycles of 95 °C for 5 s and 60 °C for 45 s. Each experimental replicate was measured in three technical wells. Technical-well values were averaged before statistical analysis and were not treated as independent observations. Relative mRNA abundance was calculated using the 2^−ΔΔCt^ method, with *GAPDH* used as the reference gene. Primer sequences, GenBank accessions, and amplicon sizes are listed in Table 1.

### 2.8. Western Blotting

After removal of the culture medium, cells were washed three times with pre-cooled PBS and lysed on ice for 20 min with 300 μL RIPA lysis buffer (P0013B, Beyotime, Shanghai, China) supplemented with phosphatase and protease inhibitors. The lysates were pipetted every 5 min to ensure complete lysis and then centrifuged at 12,000× *g* for 10 min at 4 °C. The supernatants were collected for protein quantification.

Protein concentrations were determined using a BCA protein assay kit (ZJ101, Epizyme, Shanghai, China). Equal amounts of protein were mixed with 5× protein loading buffer (P0015L, Beyotime, Shanghai, China) and denatured at 100 °C for 10 min. Proteins were separated by 10% SDS-PAGE (1610183, Bio-Rad, Hercules, CA, USA) and transferred onto PVDF membranes (IPVH00010, Millipore, Burlington, MA, USA) at 100 V for 1 h 30 min.

The membranes were blocked with 5% skimmed milk (P0216, Beyotime, Shanghai, China) in TBS-T for 1 h at room temperature and then incubated overnight at 4 °C with the following primary antibodies: anti-QKI (Abcam, Cambridge, UK, EPR7306, 1:5000), anti-Cavin3 (Proteintech, Rosemont, IL, USA, 16250-1-AP, 1:3000), anti-Adiponectin (Proteintech, 83961-4-RR, 1:2000), anti-C/EBPα (Proteintech, 29388-1-AP, 1:3000), anti-FABP4 (Proteintech, 12802-1-AP, 1:10000), anti-PPARγ (Proteintech, 16643-1-AP, 1:5000), and anti-GAPDH (Proteintech, 81640-5-RR, 1:5000).

After primary-antibody incubation, membranes were washed three times in TBS-T for 5 min and incubated with IRDye 800CW goat anti-rabbit IgG (H+L) secondary antibody (926-32211, LI-COR Biosciences, Lincoln, NE, USA; 1:10,000) for 1 h at room temperature in the dark. Membranes were scanned using an Odyssey infrared imaging system (LI-COR Biosciences, Lincoln, NE, USA). Band intensities were quantified using ImageJ 1.54s (National Institutes of Health, Bethesda, MD, USA) and normalized to GAPDH. Uncropped blot images with molecular-mass markers and lane labels are provided in the Supplementary Materials.

### 2.9. Full-Length Transcriptome Sequencing, Differential Expression and Functional Enrichment Analyses

ONT full-length transcriptome sequencing was performed on six samples, comprising three *QKI*-knockdown samples (KD1–KD3) and three negative-control samples (NC1–NC3). Each sequenced sample represented an independently treated culture well. Full-length cDNA libraries were prepared using the PCR-cDNA Sequencing Kit and sequenced on a PromethION platform equipped with FLO-PRO002 flow cells (Oxford Nanopore Technologies, Oxford, UK). Raw Nanopore signals in POD5/FAST5 format were basecalled using Dorado with the --no-trim option to generate FASTQ files. Reads with a mean quality score of at least 10 were classified as pass reads. Each sample yielded 12.163–12.249 Gb of pass data (7,134,478–11,957,991 reads), with mean Q scores of 23.33–23.93 and pass-read N50 values of 1095–2321 bp (Table 2). 

Long reads were filtered using NanoFilt v2.8.0, and full-length reads were identified using Pychopper v2.2.2. Between 5,330,710 and 9,529,042 full-length reads were retained per sample (74.72–80.37% of pass reads; full-length-read N50, 922–1985 bp). Full-length reads were aligned with minimap2 v2.24-r1122 to the sheep reference genome ARS-UI_Ramb_v3.0 (NCBI assembly GCF_016772045.2), producing mapping rates of 98.58–99.64% (Table 2). Consensus and nonredundant transcripts were generated/annotated using Pinfish v0.1.0, StringTie v2.2.1, and gffcompare v0.12.6.

Expression was quantified at both transcript and gene levels using Salmon v1.9.0. Differential expression between QKI-knockdown and control samples was analyzed at both levels using DESeq2 v1.34.0; features with |log_2_(fold change)| > 1 and Benjamini–Hochberg-adjusted *p* < 0.05 were considered differentially expressed. Unless otherwise specified, the differential expression results reported in this study refer to the gene-level analysis; transcript-level results are explicitly identified where applicable.

Gene Ontology (GO) and Kyoto Encyclopedia of Genes and Genomes (KEGG) enrichment analyses were performed against the Ovis aries annotation corresponding to the ARS-UI_Ramb_v3.0 reference genome. The main text focuses on categories most relevant to cellular remodeling and lipid metabolism.

### 2.10. Prediction of QKI-Binding Motifs in Cavin3 mRNA

The *Cavin3* 3′UTR sequence was obtained from the NCBI RefSeq database (accession no. XM_015101052.3). Candidate QKI-associated motifs were identified by manual sequence inspection. Briefly, the *Cavin3* 3′UTR sequence was scanned for ACTCAC-containing hexamers, and candidate regions were selected based on their similarity to the previously reported QKI response element sequence context [24].

### 2.11. RNA Immunoprecipitation

RNA immunoprecipitation (RIP) was performed using an RIP kit (Bes5101, Bersinbio, Guangzhou, China) according to the manufacturer’s instructions. After washing with PBS, adipocytes were collected using a cell scraper and centrifuged at 1000× *g* at 4 °C to obtain cell pellets. The cells were lysed in lysis buffer supplemented with protease inhibitors and RNase inhibitors and incubated on ice for 20 min, with vortexing every 5 min. DNase was added to remove genomic DNA. EDTA and EGTA were then added to terminate the reaction, and DTT was added to maintain protein stability.

The lysate was divided into three fractions: 0.8 mL for the QKI immunoprecipitation group, 0.8 mL for the IgG control group, and 0.1 mL for the Input group, which was stored at −80 °C. The QKI immunoprecipitation and IgG control samples were incubated overnight at 4 °C with 5 μg QKI antibody and IgG antibody, respectively. Pre-equilibrated Protein A/G magnetic beads were then added, and the samples were incubated at 4 °C for 1 h to capture antibody–antigen complexes.

After washing, the magnetic beads were resuspended in buffer, and RNA was extracted using the TRIzol method. Equal volumes of RNA were reverse-transcribed into cDNA, and enrichment of *Cavin3* mRNA was detected by qRT-PCR. The Input group was used for normalization, and the IgG group served as the negative control to evaluate nonspecific binding. To verify immunoprecipitation efficiency, a portion of the magnetic bead complexes was subjected to Western blotting to confirm successful enrichment of QKI protein.

### 2.12. mRNA Degradation Assay

To determine whether *QKI* regulates *Cavin3* expression by affecting mRNA stability, an mRNA degradation assay was performed using actinomycin D. After lentiviral infection, sheep preadipocytes were induced to differentiate as described above. Differentiated cells were then treated with 15 μg/mL actinomycin D and collected at 0, 4, 8, 12, and 24 h after treatment.

Total RNA was extracted at each time point, and equal amounts were reverse-transcribed for qRT-PCR. *Cavin3* mRNA remaining at each time point was normalized to the 0 h value. Because an mRNA half-life was not reported, the assay was interpreted from the normalized decay curves and between-group comparisons at the sampled time points; no t_1/2_ estimate is claimed in this study. Curves were plotted using GraphPad Prism 10 (GraphPad Software, Boston, MA, USA).

### 2.13. Glucose Consumption Assay

To evaluate the effects of *QKI* or *Cavin3* knockdown on glucose metabolism during adipogenic differentiation, sheep preadipocytes were transfected with lentiviral sh-*QKI* or si-*Cavin3* and then induced to differentiate as described above. One day before sample collection, the medium was replaced with phenol red-free, low-glucose complete medium. After 7 h of culture, 200 μL of culture supernatant was collected and centrifuged at 12,000× *g* for 5 min at 4 °C.

Culture supernatants were analyzed using a glucose assay kit based on the glucose oxidase–peroxidase microplate method (GOD-POD; KGH7010-200, KeyGEN BioTECH, Nanjing, China) according to the manufacturer’s instructions. Sample optical density values were converted to glucose concentrations using the accompanying standard curve. Glucose consumption was calculated as the difference between the initial glucose concentration in the culture medium and the residual glucose concentration in the collected supernatant, normalized to cellular protein content. Glucose consumption was used as an auxiliary metabolic endpoint rather than as a direct measure of triglyceride synthesis.

### 2.14. Statistical Analysis

Unless otherwise stated, cell-based experiments included three independently treated culture wells per group (*n* = 3), as specified in the corresponding figure legends. Each independently treated well was regarded as an experimental replicate. For qRT-PCR, three technical wells were analyzed for each experimental replicate, and their values were averaged before statistical analysis. Data are presented as the mean ± standard error of the mean (SEM). Comparisons between two groups were performed using unpaired two-sided Student’s *t*-tests, whereas comparisons among more than two groups were performed using one-way analysis of variance followed by Tukey’s multiple-comparison test. Statistical analyses were conducted using GraphPad Prism (GraphPad Software, Boston, MA, USA). Exact *p* values are reported where applicable, and *p* < 0.05 was considered statistically significant.

## 3. Results

### 3.1. QKI Knockdown Suppresses Adipogenic Differentiation and Reduces Glucose Consumption in Sheep Preadipocytes

**Figure 1:**
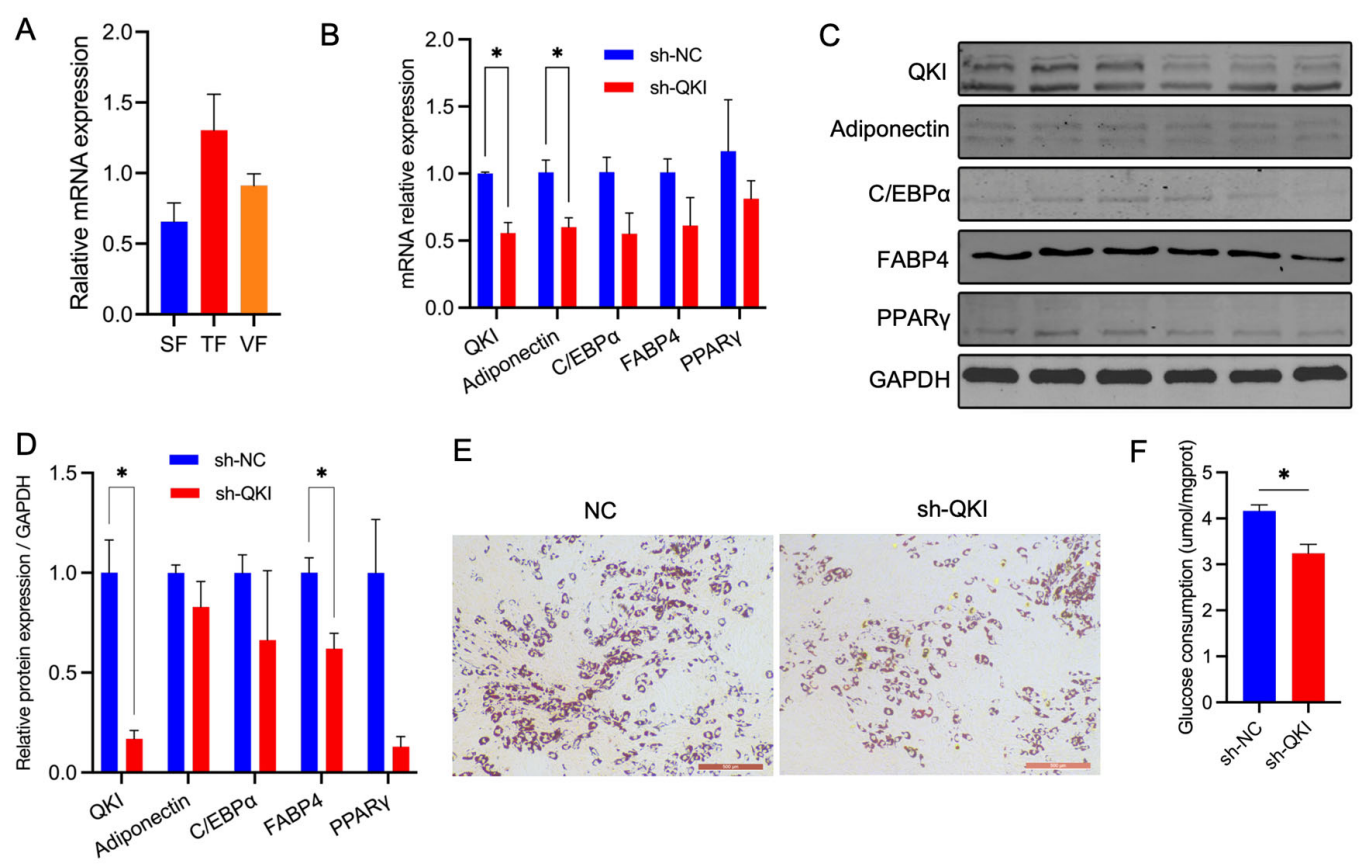
QKI knockdown suppresses adipogenic differentiation and reduces glucose consumption in sheep preadipocytes. (**A**) Relative *QKI* mRNA abundance in subcutaneous fat (SF), tail fat (TF), and visceral fat (VF). (**B**) qRT-PCR analysis of QKI, Adiponectin, C/EBPα, FABP4, and PPARγ in sh-NC and sh-QKI cells after adipogenic induction. (**C**) Representative Western blots of QKI, Adiponectin, C/EBPα, FABP4, and PPARγ, together with the GAPDH loading controls. Different target proteins were analyzed on separate membranes. For some targets, GAPDH was detected on the same membrane, whereas for others it was detected on a parallel membrane loaded with the corresponding protein samples. Uncropped blots with molecular-mass markers and lane labels are provided in the Supplementary Materials. (**D**) Densitometric analysis of target-protein abundance normalized to the corresponding GAPDH signal. (**E**) Representative Oil Red O staining images. Scale bar, 500 μm. (**F**) Glucose consumption. Data are presented as the mean ± SEM (*n* = 3 independently treated wells per group). * *p* < 0.05.

*QKI* mRNA was detected in subcutaneous fat (SF), tail fat (TF), and visceral fat (VF). Among the examined adipose tissues, *QKI* showed relatively high mRNA abundance in tail fat (Figure 1A). This expression pattern was used as a preliminary screening observation and provided an initial rationale for prioritizing *QKI* for functional investigation in sheep preadipocytes.

*QKI* mRNA was lower in sh-*QKI* than in sh-NC cells, confirming knockdown (Figure 1B; *p* = 0.0264). Among the measured adipogenic transcripts, *adiponectin* was also lower (*p* = 0.0381), whereas *C/EBPα* (*p* = 0.0802), *FABP4* (*p* = 0.1887), and *PPARγ* (*p* = 0.4596) were not significantly different. QKI protein abundance was lower after knockdown (*p* = 0.0305), and FABP4 protein abundance was also lower (*p* = 0.0238), whereas adiponectin, C/EBPα, and PPARγ protein abundances were not significantly different (Figure 1C,D).

Representative Oil Red O images showed less staining in QKI-knockdown cultures after adipogenic induction (Figure 1E). Because staining was not quantified, this observation is interpreted qualitatively. Glucose consumption, used as an auxiliary metabolic endpoint, was lower in sh-QKI cells (Figure 1F; *p* = 0.0247). Together, these findings indicate that QKI knockdown attenuated adipogenic differentiation-associated changes in sheep preadipocytes.

### 3.2. Transcriptome Analysis Identifies Cavin3 as a Candidate Downstream Target of QKI

**Figure 2:**
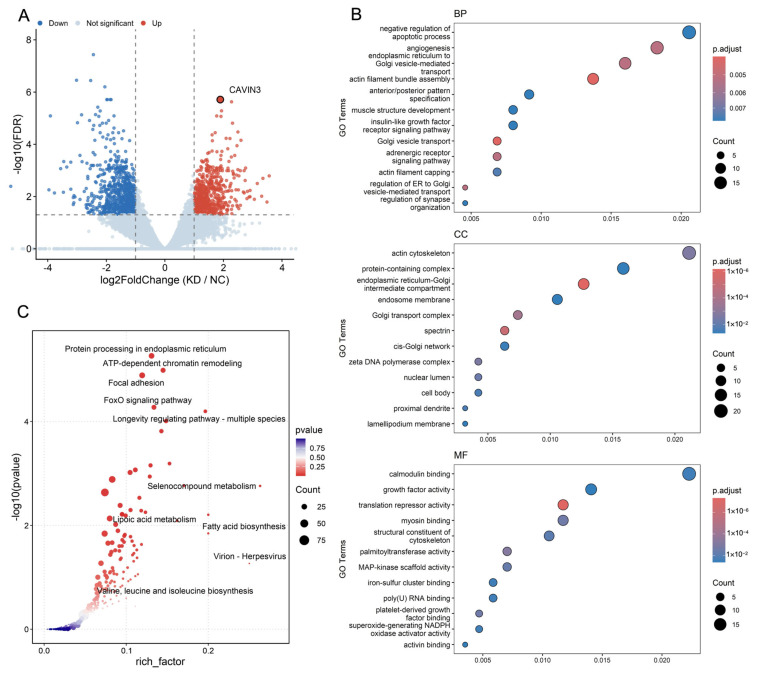
ONT full-length transcriptome sequencing identifies Cavin3 as a candidate downstream target of QKI. (**A**) Volcano plot of differentially expressed genes between three QKI-knockdown samples and three control samples. Red, blue, and gray points denote upregulated, downregulated, and nonsignificant genes, respectively. The vertical dashed lines indicate the log2 fold change thresholds (|log2FC| = 1), and the horizontal dashed line indicates the statistical significance threshold (FDR = 0.05). (**B**) Gene Ontology (GO) enrichment analysis. Dot size indicates gene count and color indicates adjusted *p* value. A full-size version of the GO enrichment plot is provided in Supplementary Figure S2. (**C**) Kyoto Encyclopedia of Genes and Genomes (KEGG) pathway enrichment analysis. Dot size indicates gene count and color indicates nominal *p* value. Three independently treated wells per group were sequenced.

ONT full-length transcriptome sequencing compared three QKI-knockdown samples with three control samples. Gene-level analysis identified 1373 differentially expressed genes (648 upregulated and 725 downregulated in the report-defined KD-versus-NC contrast), and transcript-level analysis identified 1458 differentially expressed transcripts (708 upregulated and 750 downregulated). *CAVIN3* was upregulated in KD samples at both the gene level (log_2_ fold change = 1.893; adjusted *p* = 1.94 × 10^−6^) and transcript level (log_2_ fold change = 1.897; adjusted *p* = 4.01 × 10^−6^) (Figure 2A).

GO terms relevant to the observed cellular phenotype included actin-cytoskeleton organization, vesicle transport, endoplasmic-reticulum organization, transmembrane transport, and insulin-like growth-factor receptor signaling (Figure 2B). The complete GO enrichment results are provided in Supplementary Material S2. KEGG categories included focal adhesion, FoxO signaling, endoplasmic-reticulum protein processing, and fatty-acid biosynthesis (Figure 2C). The complete KEGG enrichment results are provided in Supplementary Material S3. These enrichment analyses were used to prioritize candidate pathways and genes. *Cavin3* was selected for subsequent functional and mechanistic investigation based on the ONT results and its reported roles in caveolar function and adipogenic regulation.

### 3.3. QKI Is Associated with Cavin3 mRNA and Affects Its Stability

**Figure 3:**
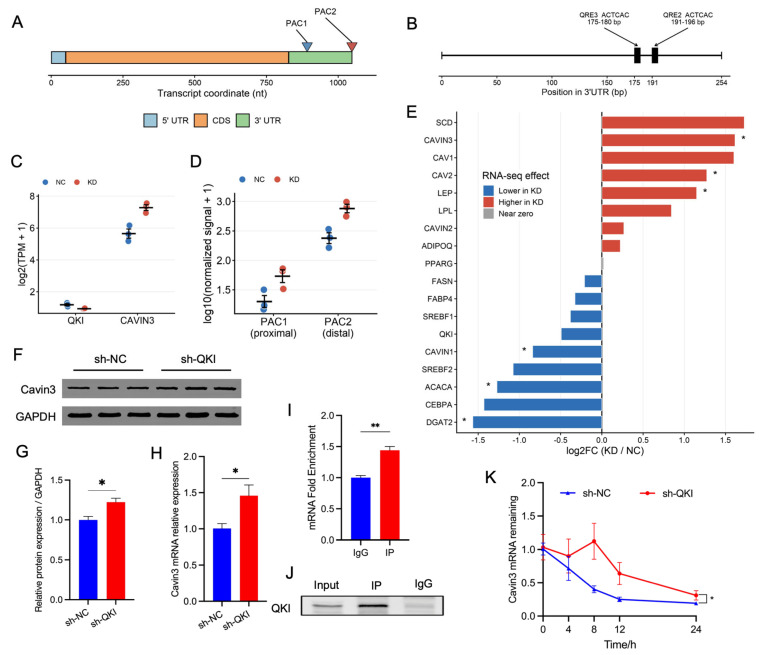
QKI is associated with Cavin3 mRNA and affects its stability. (**A**) Cavin3 transcript schematic showing the 5′UTR, coding sequence (CDS), 3′UTR, and two candidate regions. (**B**) Two predicted ACTCAC-containing regions at positions 175–180 and 191–196 of the Cavin3 3′UTR. (**C**) ONT-derived QKI and Cavin3 abundance. (**D**) Sequence context surrounding the two candidate ACTCAC-containing regions. (**E**) ONT-derived changes in selected caveolae- and adipogenesis-related features. (**F**,**G**) Representative Cavin3 Western blot and densitometry after QKI knockdown. Uncropped blots with molecular-mass markers and lane labels are provided in the Supplementary Materials. (**H**) Cavin3 qRT-PCR after QKI knockdown. (**I**) RIP-qPCR enrichment of Cavin3 mRNA in QKI versus IgG immunoprecipitates; enrichment demonstrates association under the assay conditions but not binding to a specific predicted motif. (**J**) Western blot confirmation of QKI immunoprecipitation. (**K**) Cavin3 mRNA remaining after actinomycin D treatment. Data are presented as the mean ± SEM (*n* = 3 independently treated wells per group). For the ONT-derived panels, three independently treated wells per group were sequenced. Unpaired two-sided Student’s *t*-tests were used for the indicated between-group comparisons; * *p* < 0.05 and ** *p* < 0.01.

We next assessed whether *QKI* was associated with post-transcriptional regulation of *Cavin3*. Sequence analysis nominated two ACTCAC-containing regions at positions 175–180 and 191–196 of the *Cavin3* 3′UTR as putative *QKI*-associated regions (Figure 3A,B).

ONT full-length transcriptome sequencing data showed that *Cavin3* expression was increased after *QKI* knockdown (Figure 3C). Sequence analysis identified two candidate QKI-associated regions within the Cavin3 3′UTR (Figure 3D). In addition, ONT full-length transcriptome sequencing data-based expression profiling showed that several caveolae- and adipogenesis-related genes were altered after QKI knockdown. *Cavin3*, *Cav1*, *Cav2*, *SCD*, *LEP*, and *LP* were upregulated, whereas several adipogenic and lipid metabolism-related genes, including *ADIPOQ*, *PPARG*, *FASN*, *FABP4*, *SREBF1*, *SREBF2*, *ACACA*, *CEBPA*, and *DGAT2*, were downregulated (Figure 3E).

The upregulation of Cavin3 after QKI knockdown was further confirmed at both the protein and mRNA levels. Western blot analysis showed that Cavin3 protein abundance was significantly increased in the sh-QKI group compared with the sh-NC group (Figure 3F,G; *p* = 0.0262). qRT-PCR analysis also confirmed that Cavin3 mRNA abundance was significantly increased after QKI knockdown (Figure 3H; *p* = 0.0491).

To examine the association between *QKI* and *Cavin3* mRNA, RNA immunoprecipitation followed by qRT-PCR was performed. *Cavin3* mRNA was significantly enriched in the QKI immunoprecipitation group compared with the IgG control (Figure 3I; *p* = 0.0032), indicating that Cavin3 mRNA was associated with the QKI-containing ribonucleoprotein complex. Western blot analysis of the immunoprecipitated complexes confirmed successful enrichment of QKI protein in the IP fraction, supporting the reliability of the RIP assay (Figure 3J).

To determine whether *QKI* affects *Cavin3* mRNA stability, cells were treated with actinomycin D, and the remaining Cavin3 mRNA abundance was measured at different time points. Compared with the sh-NC group, *QKI* knockdown resulted in a slower loss of *Cavin3* mRNA and higher normalized *Cavin3* mRNA abundance during the observation period (Figure 3K). These findings support an association between *QKI* and *Cavin3* mRNA and are consistent with *QKI* contributing to the post-transcriptional regulation of *Cavin3* mRNA stability.

### 3.4. Cavin3 Knockdown Promotes Adipogenesis, and Combined Knockdown Supports Its Role Downstream of QKI

**Figure 4:**
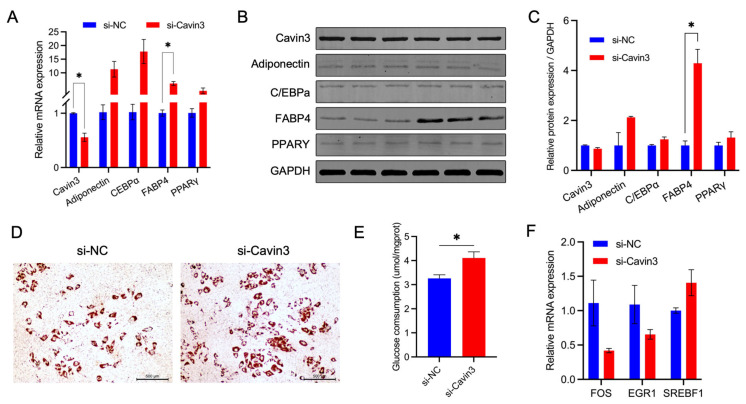
*Cavin3* knockdown promotes adipogenic differentiation and glucose consumption in sheep preadipocytes. (**A**) qRT-PCR analysis of *Cavin3*, *Adiponectin*, *C/EBPα*, *FABP4*, and *PPARγ* after *Cavin3* knockdown. *ADIPOQ*, *CEBPA*, and *PPARG* were not significantly different. (**B**,**C**) Representative Western blots and densitometric analyses. Target-protein abundance was normalized to the corresponding GAPDH loading control. (**D**) Representative Oil Red O images; staining was not quantified. Scale bar, 500 μm. (**E**) Glucose consumption. (**F**) *FOS*, *EGR1*, and *SREBF1* mRNA abundance; none differed significantly between groups, and these measurements do not establish ERK or AKT pathway activity. Data are presented as the mean ± SEM (*n* = 3 independently treated wells per group). Unpaired two-sided Student’s *t*-tests were used for the indicated between-group comparisons; * *p* < 0.05. Uncropped blots with molecular-mass markers and lane labels are provided in the supplied Supplementary Materials.

To determine whether *Cavin3* participates in sheep preadipocyte differentiation, siRNA-mediated *Cavin3* knockdown was performed. qRT-PCR analysis showed that *Cavin3* mRNA abundance was significantly reduced in the si-*Cavin3* group compared with the si-NC group, confirming efficient *Cavin3* knockdown (Figure 4A; *p* = 0.0046). After adipogenic induction, *FABP4* mRNA abundance was significantly increased following *Cavin3* knockdown (*p* = 0.0022), whereas *Adiponectin*, *C/EBPα*, and *PPARγ* mRNA abundances were not significantly different between the groups (Figure 4A).

Western blot analysis showed that Cavin3 protein abundance was reduced after siRNA transfection. FABP4 protein abundance was significantly increased following Cavin3 knockdown (*p* = 0.0049), whereas Adiponectin, C/EBPα, and PPARγ protein abundances were not significantly different (Figure 4B,C). Representative Oil Red O staining images showed greater staining in the si-Cavin3 group than in the si-NC group (Figure 4D). In addition, glucose consumption was significantly increased after Cavin3 knockdown (Figure 4E; *p* = 0.0462).

To explore potential signaling changes associated with *Cavin3* knockdown, the expression of ERK/AKT pathway-related genes was examined using qRT-PCR. *FOS* and *EGR1* mRNA abundances were numerically lower, whereas *SREBF1* mRNA abundance was numerically higher, in the si-*Cavin3* group than in the si-NC group, although these differences did not reach statistical significance (Figure 4F). These coordinated directional changes suggest that *Cavin3* knockdown may influence ERK/AKT-associated transcriptional responses in sheep preadipocytes. Further assessment of *ERK* and *AKT* phosphorylation will be needed to clarify the involvement of these signaling pathways.

To further examine whether *Cavin3* contributes to the phenotype associated with *QKI* depletion, an independent combined-knockdown experiment was performed. qRT-PCR confirmed that *QKI* and *Cavin3* mRNA abundances were significantly reduced in the double-knockdown (DKD) group compared with the matched negative-control (NC) group (Supplementary Figure S1A). Following adipogenic induction, *ADIPOQ*, *FABP4*, and *PPARG* mRNA abundances were significantly increased in DKD cells, whereas *CEBPA* mRNA abundance was not significantly different between the groups. Representative Oil Red O staining images showed qualitatively greater lipid-droplet accumulation in the DKD group than in the NC group (Supplementary Figure S1C). Glucose consumption was not significantly different between the DKD and NC groups (Supplementary Figure S1B). Because this experiment was performed independently from the original *QKI* single-knockdown experiment, these findings were interpreted as evidence consistent with *Cavin3* contributing to the adipogenic phenotype associated with QKI depletion, rather than as a quantitative assessment of phenotypic rescue.

Together, these findings indicate that *Cavin3* knockdown increased *FABP4* mRNA and protein abundance and glucose consumption and was accompanied by qualitatively greater Oil Red O staining, supporting a negative association between *Cavin3* and sheep preadipocyte differentiation.

## 4. Discussion

The principal finding of this study is that *QKI* and *Cavin3* exerted opposing effects on sheep preadipocyte differentiation. *QKI* knockdown reduced selected adipogenic markers, lipid staining, and glucose consumption, whereas *Cavin3* knockdown enhanced several adipogenesis-associated endpoints. *Cavin3* mRNA was enriched in QKI immunoprecipitates, its abundance increased after QKI knockdown, and its decay was delayed following transcriptional inhibition. Together, these observations support a model in which *Cavin3* contributes to, but may not fully account for, the adipogenic effects associated with *QKI*.

*QKI* regulates RNA splicing, localization, translation, and stability in a transcript- and cell-context-dependent manner [13,14,15,16,17]. Its established roles in differentiation and adipose metabolism provided the rationale for examining sheep preadipocytes [17,18]. *QKI* was detected in all three adipose depots examined, with relatively high numerical abundance in tail fat. This descriptive pattern provided an initial rationale for investigating *QKI* in sheep preadipocytes, although depot-specific functions remain to be established.

The opposing changes in glucose consumption after *QKI* and *Cavin3* knockdown were consistent with their corresponding adipogenic phenotypes. Nevertheless, glucose consumption is an auxiliary metabolic endpoint, and the Oil Red O images provide qualitative rather than quantitative evidence of lipid accumulation. Future studies should incorporate quantitative lipid staining, triglyceride measurements, and metabolic-flux analyses.

The transcriptome analysis identified changes in cellular-remodeling and lipid-metabolism categories and was used to prioritize *Cavin3* for validation. Enrichment results are hypothesis-generating and do not establish activation of the named pathways.

*Cavin3* contributes to caveolar organization and signaling [19,20,21]. In mouse 3T3-L1 cells and human adipose-derived stem cells, *Cavin3* silencing retarded adipogenesis, whereas overexpression accelerated it through a mechanism involving TACE-mediated Pref-1 shedding [21]. A porcine study likewise reported that *Cavin3* promoted preadipocyte differentiation and ERK phosphorylation [22]. These findings differ from those obtained in the present sheep cultures, in which *Cavin3* knockdown increased *FABP4* abundance, qualitative lipid staining, and glucose consumption. The discrepancy may reflect differences in species, adipose depot, developmental stage, cell-isolation procedure, or differentiation-induction protocol. These contrasting findings also suggest that the role of *Cavin3* in adipogenesis may be context-dependent rather than universally positive or negative.

The proposed post-transcriptional link is biologically plausible. *QKI* recognizes *QKI* response elements in target RNAs [24], and QKI-6 has previously been shown to reduce the stability of actin-interacting protein-1 mRNA through a 3′UTR response element [25]. In the present study, QKI RIP enriched *Cavin3* mRNA, *QKI* depletion increased *Cavin3* mRNA and protein abundance, and QKI depletion was associated with slower Cavin3 mRNA decay, with a higher remaining mRNA level observed at the final sampled time point. These observations are consistent with *QKI*-associated destabilization of *Cavin3* mRNA. Nevertheless, RIP does not prove direct contact between QKI and the two candidate regions within the *Cavin3* 3′UTR. Reporter assays using wild-type and motif-mutant *Cavin3* 3′UTRs, together with an orthogonal binding assay, are needed to establish motif-specific direct regulation.

The combined-knockdown experiment provided additional functional support for *Cavin3* acting downstream of *QKI*. Simultaneous depletion of *QKI* and *Cavin3* increased *ADIPOQ*, *FABP4*, and *PPARG* mRNA abundance and enhanced qualitative lipid staining relative to the matched control, suggesting that the reduction in *Cavin3* partially offset the phenotype associated with *QKI* depletion. Because this experiment was performed independently from the original single-knockdown experiment, the magnitude of rescue could not be quantified directly. A same-batch factorial design will be required to define the functional interaction between *QKI* and *Cavin3* more precisely.

*FOS* and *EGR1* are immediate-early genes whose expression can reflect MAPK signaling dynamics [26]. *Cavin3* knockdown produced coordinated numerical changes in pathway-associated transcripts, with lower *FOS* and *EGR1* and higher *SREBF1* abundance, although none of these differences reached statistical significance. These directional changes provide a preliminary basis for examining whether *Cavin3* influences MAPK/ERK- and AKT-associated signaling during adipogenesis. Measurements of phosphorylated and total ERK and AKT, ideally combined with pathway perturbation, will be necessary to test this possibility in sheep preadipocytes.

This study has several limitations. First, although the independent combined-knockdown experiment supports a functional relationship between *QKI* and *Cavin3*, a same-batch factorial design is needed to quantify their functional interaction. Second, RIP-qPCR and mRNA-decay analyses support an association between *QKI* and *Cavin3* mRNA stability but do not establish direct binding to the predicted 3′UTR regions. Third, lipid accumulation was evaluated qualitatively, and glucose consumption was used as an auxiliary metabolic endpoint; quantitative triglyceride and metabolic-flux measurements will therefore be required. Finally, the pathway analysis was limited to selected transcripts, and the in vivo and depot-specific roles of the *QKI*-*Cavin3* relationship remain to be determined.

## 5. Conclusions

*QKI* knockdown impaired selected features of sheep preadipocyte differentiation, whereas Cavin3 knockdown enhanced several adipogenesis-associated endpoints. The combined-knockdown findings further support a functional contribution of Cavin3 to the phenotype associated with *QKI* depletion. QKI RIP enrichment and the slower decay of *Cavin3* mRNA after *QKI* knockdown are consistent with post-transcriptional regulation of *Cavin3* by *QKI*. Collectively, these findings support a model in which *QKI* promotes sheep preadipocyte differentiation by reducing *Cavin3* mRNA stability. However, because combined knockdown restored adipogenic differentiation but did not fully rescue glucose consumption, *QKI* may regulate glucose metabolism through additional targets independent of *Cavin3*.

## Figures and Tables

**Table 1:** Primer sequences used for qRT-PCR.

Gene	GenBank Accession	Forward Primer (5′→3′)	Reverse Primer (5′→3′)	Product (bp)
*QKI*	XM_027972642.2	AAGAGAGCCGTTGAGGAAGTGAAG	TCTGTAGGTGCCGTTGAGAATCG	111
*Cavin3*	XM_015101052.3	CACGTTCTGCTCTTCAAGGAGG	CTCGTCTGAGCTCTCTTCAGC	129
*PPARG*	NM_001100921.1	AGACGACAGACAAATCACCGT	CGAAACTGACACCCCTGGAA	145
*CEBPA*	NM_001308574.1	GAGTTCCTGGCCGACCTGTTC	AAAGTCGTTGCCGCCTCCTG	87
*FABP4*	NM_001114667.1	TGACAGGAAAGTCAAGAGCATC	TCCAGCACCAGCTTATCATCC	120
*ADIPOQ*	NM_001308565.1	ATCCCCGGGCTGTACTACTT	CTGGTCCACGTTCTGGTTCT	129
*EGR1*	NM_001142506.1	CTGGAGGAGATGATGCTGCTGAG	TGCTGCTGCTGCTGCTACC	94
*FOS*	NM_001166182.1	AAGGAGAATCCGAAGGGAAAGG	TAGTTGGTCTGTCTCCGCTT	100
*SREBF1*	XM_027974784.2	TCCGACACCACCAGCATCAAC	AAGAGCACCAGCAGCCCATTC	131
*GAPDH*	NM_001190390.1	TCGGAGTGAACGGATTTGGC	AGGGGTCATTGATGGCAACG	93

**Table 2:** ONT sequencing and alignment metrics reported for the six sequenced samples.

Sample	Pass Data (Gb)	Pass Reads	Mean Q	Read N50 (bp)	Full-Length Reads	Mapping Rate
KD1	12.249	7,566,154	23.91	2176	5,790,762	99.34%
KD2	12.206	7,252,658	23.84	2321	5,499,421	99.64%
KD3	12.163	7,134,478	23.93	2304	5,330,710	99.48%
NC1	12.232	11,245,012	23.52	1216	9,037,743	98.88%
NC2	12.217	11,957,991	23.33	1095	9,529,042	98.58%
NC3	12.164	8,088,758	23.84	1932	6,090,089	99.31%

## Data Availability

The data supporting the findings of this study are available from the corresponding author upon reasonable request.

## Supplementary Materials

The following supporting information can be downloaded at: https://www.mdpi.com/article/10.3390/ani16152441/s1.
