# Supplementary material for: QKI Promotes Sheep Preadipocyte Differentiation by Reducing Cavin3 mRNA Stability
Source: Animals (Basel). 2026 Aug 6;16(15):2441. doi: 10.3390/ani16152441 (PMC13463844; doi:10.3390/ani16152441)

# QKI Promotes Sheep Preadipocyte Differentiation by Reducing Cavin3 mRNA Stability

Zicheng Li [1,2], Changsong Xu [1,2], Bokang Shan [1,2], Yuan Wang [1,2], Wangyang Qin [1,2], Lei Xia [3], Liying Qiao [1,2], Wenzhong Liu [1,2], Yangyang Pan [1,2,3,*]

**Supplementary Table S1.** Sequences of short hairpin RNAs targeting QKI

| Oligo name | Sequence |
|---|---|
| QKI-sh-F1 | GATCCGAGTGCAGAATTGCCTGATGCTGTGTTCAAGAGACACAGCATCAG GCAATTCTGCACTCTTTTTTG |
| QKI-sh-R1 | AATTCAAAAAA GAGTGCAGAATTGCCTGATGCTGTGTCTCTT GAA CACAGCATCAGGCAATTCTGCACTCG |
| QKI-sh-F2 | GATCCCAGCTTGAAGCAGAAACAGGATGTATTCAAGAGATACATCCTGTTT CTGCTTCAAGCTGTTTTTTG |
| QKI-sh-R2 | AATTCAAAAAA CAGCTTGAAGCAGAAACAGGATGTATCTCTT GAA TACATCCTGTTTCTGCTTCAAGCTGG |
| QKI-sh-F3 | GATCCCGGCACCTACAGAGATGCCAACATTTTCAAGAGAAATGTTGGCATC TCTGTAGGTGCCGTTTTTTG |
| QKI-sh-R3 | AATTCAAAAAA CGGCACCTACAGAGATGCCAACATTTCTCTT GAA AATGTTGGCATCTCTGTAGGTGCCGG |
| NC-F | GATCCGATGAAATGGGTAAGTACATTCAAGAGATGTACT TACCCATTTCATCTTTTTTG |
| NC-R | AATTCAAAAAAGATGAAATGGGTAAGTACATCTCTTGA ATGTACTTACCCATTTCATCG |

**Supplementary Table S2**. siRNA sequences targeting Cavin3

| siRNA Names | Sequences (5'→3') | |
|---|---|---|
| | Sense | Antisense |
| Cavin3-1 | GCCCGAAGCCGAAGCUGAATT | UUCAGCUUCGGCUUCGGGCTT |
| Cavin3-2 | CGGUGGUGACCCUGCUGGATT | UCCAGCAGGGUCACCACCGTT |
| Cavin3-3 | CCGGGUUGGAGAAGGUACATT | UGUACCUUCUCCAACCCGGTT |
| Negative Control | UUCUCCGAACGUGUCACGUTT | ACGUGACACGUUCGGAGAATT |

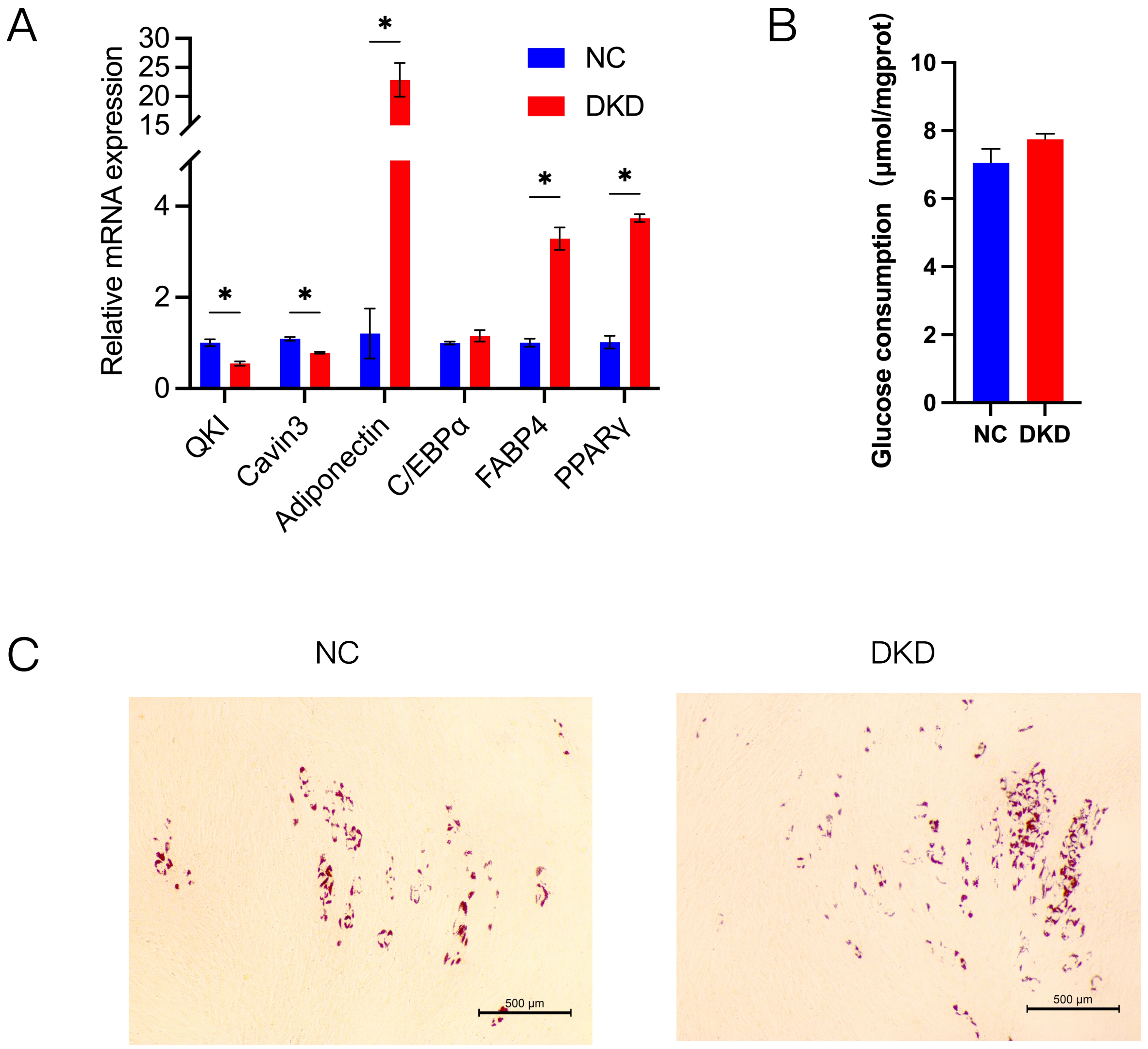


**Supplementary Figure S1.** Effects of combined QKI and Cavin3 knockdown on adipogenic differentiation in sheep preadipocytes.

(A) qRT–PCR analysis of QKI, Cavin3, and adipogenic marker genes, including ADIPOQ, CEBPA, FABP4, and PPARG, in the negative control (NC) and QKI/Cavin3 double–knockdown (DKD) groups after adipogenic induction. (B) Glucose consumption in NC and DKD cells after adipogenic induction. (C) Representative Oil Red O staining images showing lipid droplet accumulation in NC and DKD cells. Scale bar, 500 μm. Data are presented as the mean ± SEM from at least three independent biological replicates. Statistical significance was determined using an unpaired Student's t–test. $P < 0.05$.

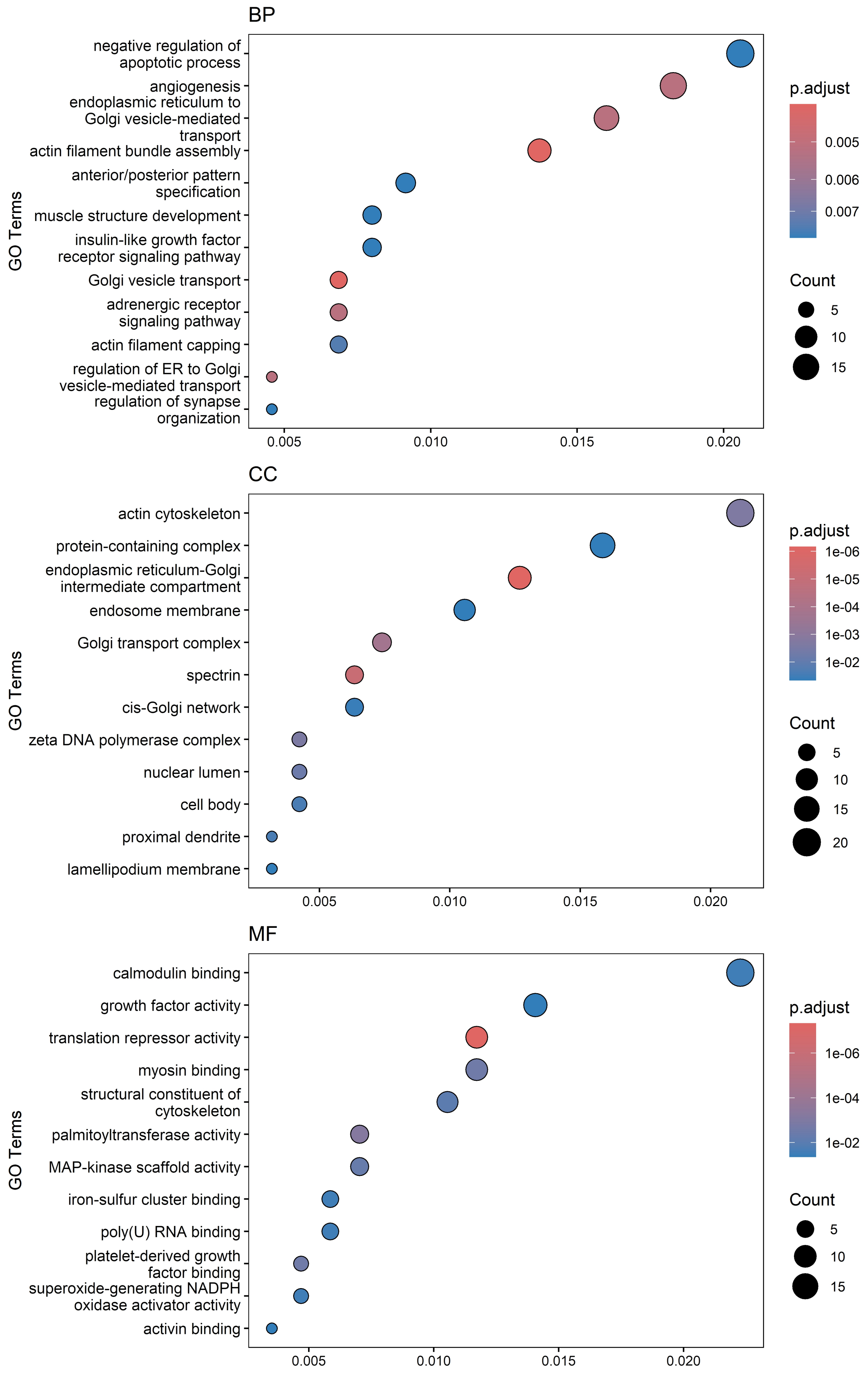


**Supplementary Figure S2.** Full-size Gene Ontology (GO) enrichment plot corresponding to Figure 2B.

# Uncropped Western blot images of adipogenic marker proteins in negative control and QKI-knockdown sheep preadipocytes

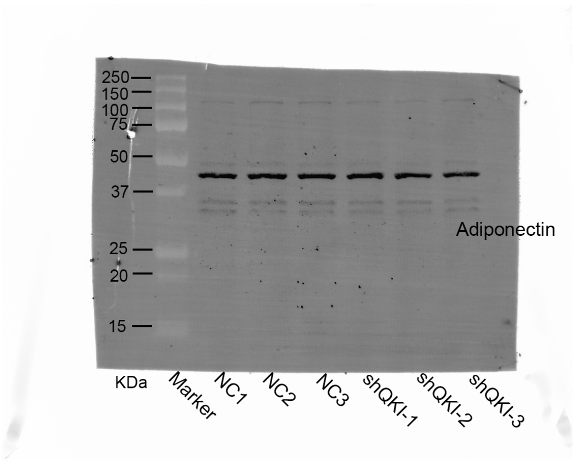


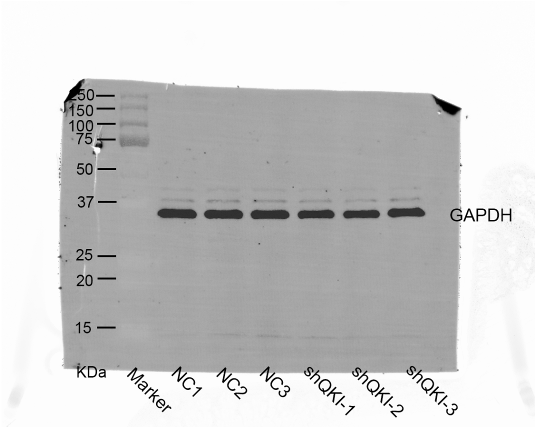


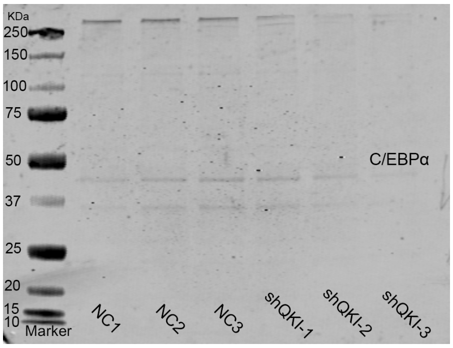


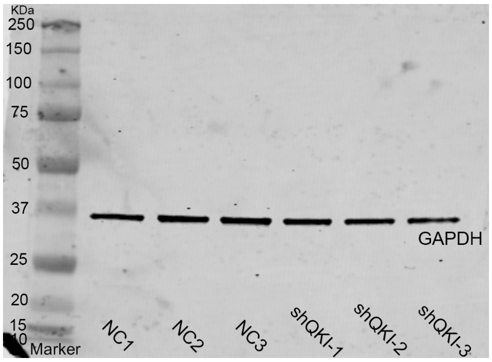


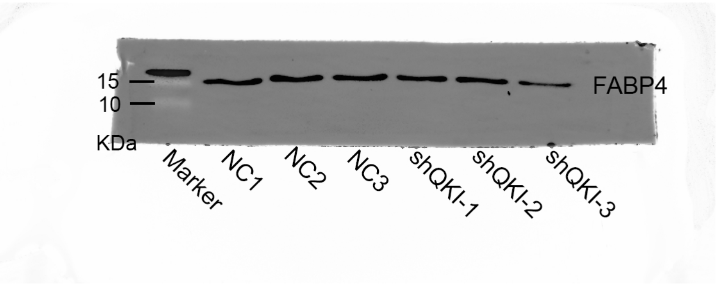


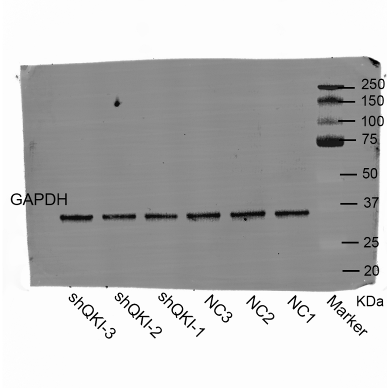


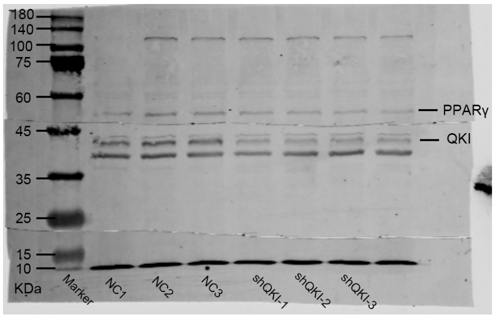


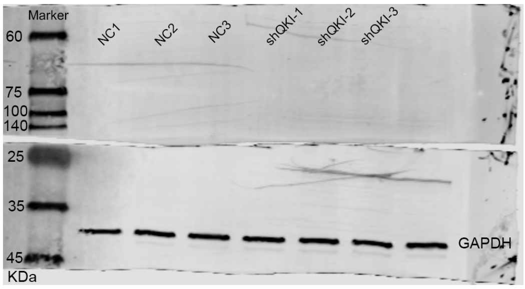

# Uncropped Western blot images of adipogenic marker proteins in negative control and CAVIN3-knockdown sheep preadipocytes

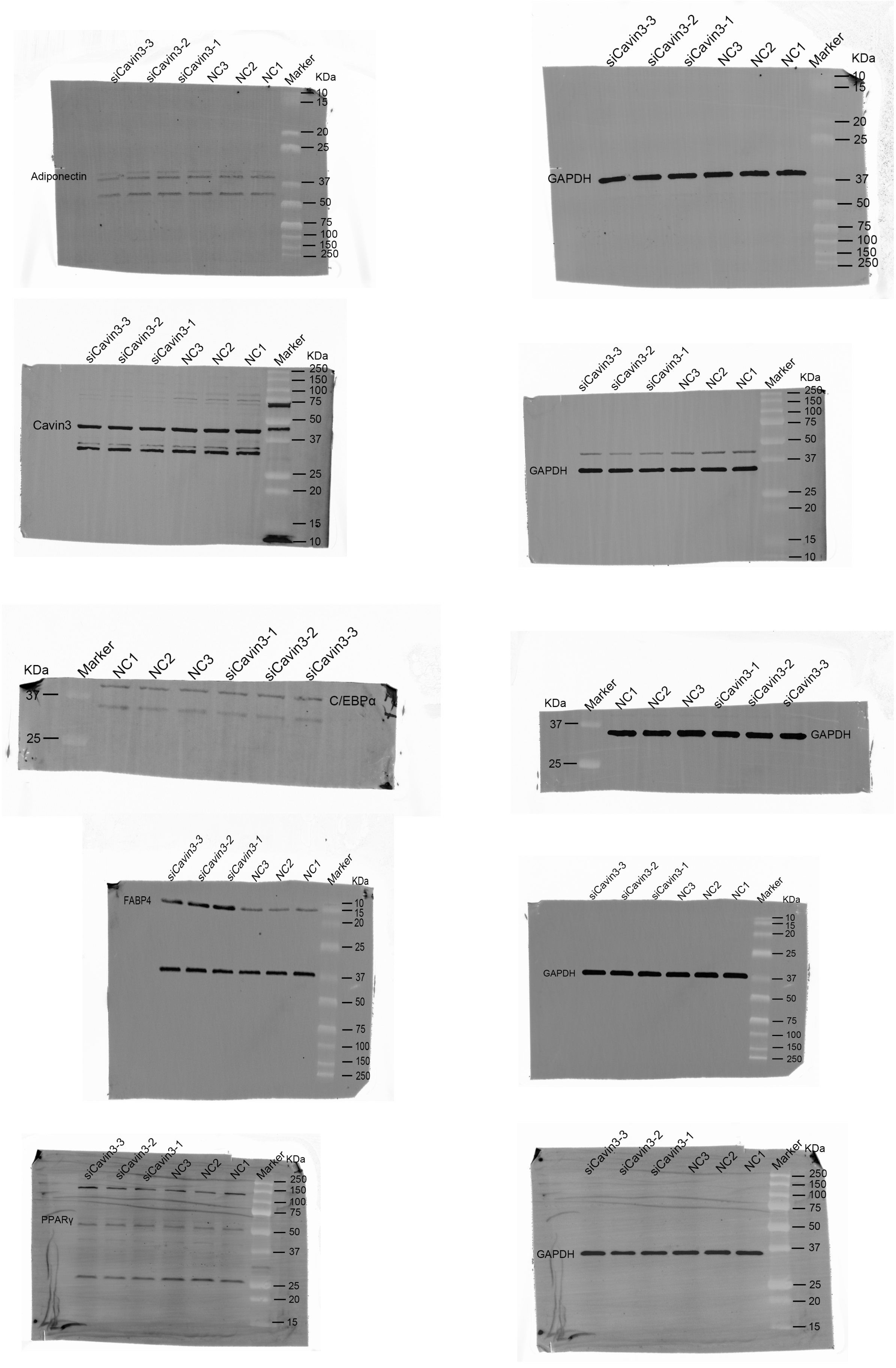

Supplement: Supplementary file 1 [file animals-16-02441-s001.zip › Supplementary Materials S1.pdf]
